# Supplementary material for: Getting More Out of Biomedical Documents with GATE's Full Lifecycle Open Source Text Analytics
Source: PLoS Comput Biol. 2013 Feb 7;9(2):e1002854. doi: 10.1371/journal.pcbi.1002854 (PMC3567135; doi:10.1371/journal.pcbi.1002854)
Supplement: Dataset S1 — GATE software. Dataset S1 bundled with this paper contains a distribution of GATE (or see http://gate.ac.uk/download/). (TGZ) [file pcbi.1002854.s001.tgz › plos-gate/doc/usecases.html]

GATE 2 usecases


## Use cases

There are these categories of work on Software
Architecture for Language Engineering (SALE):

1. **Processing resources**
   1. *Locating, loading and initialising components from local and non-local
      machines.*
   2. *Executing processing components, serially or in parallel.*
   3. *Representing information about components.*
   4. *Factoring out commonalities amongst components.*
2. **Language Resources, corpora and annotation**
   1. *Accessing data components.*
   2. *Managing collections of documents (including recordings) and their
      formats.*
   3. *Representing information about text and speech.*
   4. *Representing information about language.*
   5. *Indexing and retrieving information.*
3. **Methods and applications**
   1. *Method support*
      - *FST, unification and statistics over information.*
      - *Comparing different versions of information.*
   2. *Application issues*
      - *Storing information.*
      - *Deployment and embedding.*
   3. *Development issues*
      - *Interoperation with other infrastructures.*
      - *Viewing and editing data components and information.*
      - *UI access to architectural facilities (development environments).*

The rest of this page presents use cases for these categories.

## General desiderata

---

**Use case 1**: LE research and development

**Goal**:
To support LE R&D workers producing software and performing
experiments.

**Description**:
During design, developers use the architectural component of SALE
for guidance on the overall shape of the system.
During development they use the framework
for implementations of the architecture, and of commonly occuring tasks.
The development environment is used for convenient ways of
exploiting the framework and of accessing common tasks.
For deployment the framework is available independently of the development
environment and can be embedded in other applications.

---

**Use case 2**: Documentation, maintenance, and support

**Goal**:
To document, maintain and support the architecture.

**Description**:
Without adequate documentation of its facilities an architecture is next to
useless. Without bug fixes and addition of new features to meet changing
requirements it will not evolve and fall into disuse. Without occasional
help from experts users will learn more slowly than they could.

---

**Use case 3**: Localisation and internationalisation

**Goal**:
To allow the use of the architecture in and for different languages.

**Description**:
Users of the architecture need to be able to have menus and at least some of
the documentation in a familiar language, and they need to be able to build
applications which process and display virtually any human language.

---

**Use case 4**: Software development good practice

**Goal**:
To promote good software engineering in LE development.

**Description**:
We can derive a number of
general desiderata for SALEs on the basis that they are used for software
development. In common with other software developers, SALE users need
extensibility;
interoperability;
openness;
explicit design documentation in appropriate modelling languages;
graphical development environments;
usage examples, or patterns.

---

**Use case 5**: Framework requirements

**Goal**:
To exploit the benefits of the framework.

**Description**:
Some general requirements for frameworks:

Orthogonality of elements: a user shouldn't have to learn everything in order
to use one thing.

Availability of abstractions at different levels of complexity: a user should
be able to do something basic in a simple fashion, but also be able to fiddle
under the hood in a more complex scenario if necessary.

---

## Components, PRs and LRs

---

### Locating, loading and initialising components

---

**Use case 6**: Locate and load components

**Goal**:
To discover components at runtime, load and initialise them.

**Description**:
R&D developers create LR and PR components and reuse those created by others.
Experimenters, students and teachers use components provided for them. Systems
administrators install components. Applications developers embedd components
in their systems. The set of components required in different cases is dynamic
and loading should be dynamic as a consequence. The SALE should find all
available components given minimal clues (perhaps a list of URLs), load them
and initialise them ready for use.

---

### Executing processing components

---

**Use case 7**: PR and LR Management

**Goal**:
To allow the building of systems from sets of components.

**Description**:
Developers need to be able to choose a subset of the available components and
wire them together to form systems. These configurations should be shareable
with other developers.

---

**Use case 8**: Distributed Processing

**Goal**:
To allow the construction of systems based on components residing on different
host computers.

**Description**:
Components developed on one computer platform are seldom easy to move to other
platforms. In order to reuse a diverse set of such components they must be
made available over the net for distributed processing. Networks are often
slow, however, so there must also be the capability to do all processing on
one machine if the component set allows.

---

**Use case 9**: Parallel Processing

**Goal**:
To allow asynchronous execution of processing components.

**Description**:
Certain tasks can be carried out in parallel in some language processing
systems. This implies that the execution of PRs should be
multithreaded and means made available for parallel execution.

---

### Representing information about components.

---

**Use case 10**: Component metadata

**Goal**:
To allow the association of structured data with LR and PR components.

**Description**:
Components are wired together with executive and task-specific
code to form experimental systems or applications. Component metadata helps
automate the wiring process, e.g. by describing the I/O contraints of the
component. To use components they have to be found: metadata can be used to
allow categorisation and description for browsing component sets.

---

### Factoring out commonalities amongst components.

---

**Use case 11**: Component commonalities

**Goal**:
To factor out commonalities between related components.

**Description**:
Where there are families of components that share certain characteristics
those commonalities should be modelled in the architecture.
For example language analyser PRs characteristically take a document as input
and add certain annotations to the document. Developers of analysers should be
able to extend a part of the model which captures this and other
characteristics.

---

### Accessing data components

---

**Use case 12**: LR access

**Goal**:
To provide uniform, simple methods for accessing data components.

**Description**:
Just as the execution of PRs should be normalised by a SALE, so access to data
components should be done in a uniform and efficient manner.

---

### Managing collections of documents their formats

---

**Use case 13**: Corpora (Language Data LRs)

**Goal**:
To manage (possibly very large) collections of documents in an efficient
manner.

**Description**:
Documents (texts and audiovisual materials)
are grouped into collections which may have data associated
with them. Operations which relate to documents should be generalisable
to collections of documents.

---

**Use case 14**: Format-Independent Document Processing

**Goal**:
To allow SALE users to use documents of various formats without
knowledge of those formats.

**Description**:
Documents can be processed independent of their formats. For example, an
IE system can get to the text in an RTF document or an HTML
document without worrying about the structure of these formats.
The structure is available for access where needed.

---

### Representing information about text and speech

---

**Use case 15**: Annotations on Documents

**Goal**:
To support theory-neutral format-independent annotation of documents.

**Description**:
Many of the data structures produced and consumed by PR components are
associated with text. Even NLG components can be viewed as producing data
structures that relate to nascent texts that become progressively better
specified, culminating in surface strings of words. See also interoperation
use case (annotation import/export to/from SGML/XML).

---

### Representing information about language

---

**Use case 16**: Data About Language LRs

**Goal**:
To support creation and maintenance of LRs that describe language.

**Description**:
Lexicons, grammars, ontologies, etc. etc. all require support tools for their
development, for example for consistency checking, browsing and so on. (Note
that this use case is potentially very large, and may fall outside of our
scope.) In addition, developers of these types of resource use tools such as
concordancers (e.g. KWIC) which should be provided by the development
environment.

---

### Indexing and retrieving information

---

**Use case 17**: Indices

**Goal**:
To cater for indexing and retrieval of diverse data structures.

**Description**:
The architecture includes data structures for annotating documents and for
associating metadata with components. These data structures need efficient
indices to make computation over large data sets tractable.

---

## Method support

---

**Use case 18**: Common algorithms

**Goal**:
To provide a library of well-known algorithms over native data structures.

**Description**:
Although infrastructure should not in general stray into open research fields,
where a particular algorithm is well-known it would be advantageous to provide
a baseline implementation. For example, finite state transduction over
annotation data structures, perhaps unification, ngram models and so on.
(This use case is not under the annotation heading because it would be
advantageous to generalise its application across other data structures and
across text itself in some cases.)

---

**Use case 19**: Data comparison

**Goal**:
To provide simple methods for comparing data structures.

**Description**:
Machine learning methods, evaluation methods and introspective methods all
need ways of comparing desired results on a particular language processing
task with the results that a set of components has produced. In some cases
this is a complex task (e.g. the comparison of MUC templates was found in some
circumstances to be NP complete!), but in many cases a simple comparison
measure based on identity is useful for a first-cut approximantion of success.
This measure can be expressed as precision/recall where appropriate.
(This use case is not under the annotation heading because it would be
advantageous to generalise its application across other data structures and
across text itself in some cases.)

---

## Application issues

---

**Use case 20**: Persistence

**Goal**:
All data structures native to the architecture should be persistent.

**Description**:
The storage of data created automatically
by components or manually by editing should be
managed by the framework. This management should be transparent to a large
degree, but must also be efficient and therefore should be amenable to
tinkering where necessary. Access control may also be provided here.

---

**Use case 21**: Deployment

**Goal**:
To allow the use of the framework in diverse contexts.

**Description**:
The framework must be available in many context in order to allow the
transfer of experimental and prototype systems from the development environment
to external applications and parts of applications. Users must be able to use
framework classes as a library, including classes of their own that are
derived from the framework classes. They should also be able to build programs
based on the framework by supplying their own executive code, and be able to
access data resources from other contexts using standard database protocols.

---

## Development issues

---

**Use case 22**: Interoperation and Embedding

**Goal**:
To enable data import from and export to other infrastructures
and embedding of components in other environments.

**Description**:
Formats and formalisms for the expression of LRs come in many shapes and
sizes. Some of these are dealt with by wrapping those formats in code that
talks the language of the SALE framework. Other, widespread formats should be
made more generally accessible via import/export filters. The prime case here
is SGML/XML.

Certain common execution environments should be catered for, such as MS
Office, OLE and Netscape Communicator.

---

**Use case 23**: Viewing and Editing

**Goal**:
To manipulate LE data structures.

**Description**:
SALEs are used to view and edit the data structures that LE systems
process. This applies to both LRs and PRs.

---

**Use case 24**: Development UI

**Goal**:
To give access to all the framework and architectural services and support
development of LE experiments and applications.

**Description**:
A large part of the story is components, which can be viewed, edited,
stored, accessed from the framework API and so on. The final element is a UI
for developers that wires all these together and gives top-level access to
storage and component management, and execution of PRs.
